# Supplementary material for: Multi‐dataset identification of innovative feature genes and molecular mechanisms in keratoconus
Source: J Cell Mol Med. 2024 Sep 19;28(18):e70079. doi: 10.1111/jcmm.70079 (PMC11412914; doi:10.1111/jcmm.70079)
Supplement: Supplementary file 2 — Material S2. [file JCMM-28-e70079-s002.zip › 7.GSEA analysis/6.SNORA74B/KEGG_GLYCOSPHINGOLIPID_BIOSYNTHESIS_GANGLIO_SERIES.html]

Details for gene set KEGG\_GLYCOSPHINGOLIPID\_BIOSYNTHESIS\_GANGLIO\_SERIES[GSEA]

|  || Dataset | SNORA74B.group.cls#h\_versus\_l |
| Phenotype | group.cls#h\_versus\_l |
| Upregulated in class | h |
| GeneSet | KEGG\_GLYCOSPHINGOLIPID\_BIOSYNTHESIS\_GANGLIO\_SERIES |
| Enrichment Score (ES) | 0.19112651 |
| Normalized Enrichment Score (NES) | 0.5288934 |
| Nominal p-value | 0.96928984 |
| FDR q-value | 1.0 |
| FWER p-Value | 1.0 |
Table: GSEA Results Summary

  

Fig 1: Enrichment plot: KEGG\_GLYCOSPHINGOLIPID\_BIOSYNTHESIS\_GANGLIO\_SERIES      
 Profile of the Running ES Score & Positions of GeneSet Members on the Rank Ordered List

  

| SYMBOL | TITLE | RANK IN GENE LIST | RANK METRIC SCORE | RUNNING ES | CORE ENRICHMENT || 1 | ST3GAL5 | na | 2284 | 0.348 | 0.1826 | Yes |
| 2 | ST6GALNAC6 | na | 8925 | 0.115 | 0.0736 | Yes |
| 3 | SLC33A1 | na | 9598 | 0.101 | 0.1260 | Yes |
| 4 | B3GALT4 | na | 10824 | 0.078 | 0.1462 | Yes |
| 5 | ST8SIA1 | na | 11894 | 0.062 | 0.1594 | Yes |
| 6 | ST6GALNAC5 | na | 12896 | 0.048 | 0.1649 | Yes |
| 7 | ST3GAL1 | na | 13468 | 0.042 | 0.1784 | Yes |
| 8 | B4GALNT1 | na | 13934 | 0.037 | 0.1911 | Yes |
| 9 | ST6GALNAC3 | na | 16535 | 0.009 | 0.1227 | No |
| 10 | HEXA | na | 17794 | -0.004 | 0.0890 | No |
| 11 | ST6GALNAC4 | na | 21165 | -0.042 | 0.0218 | No |
| 12 | GLB1 | na | 22952 | -0.065 | 0.0164 | No |
| 13 | HEXB | na | 23118 | -0.067 | 0.0596 | No |
| 14 | ST8SIA5 | na | 24259 | -0.085 | 0.0874 | No |
| 15 | ST3GAL2 | na | 31591 | -0.300 | 0.0904 | No |
Table: GSEA details [plain text format]

  

Fig 2: KEGG\_GLYCOSPHINGOLIPID\_BIOSYNTHESIS\_GANGLIO\_SERIES      
 Blue-Pink O' Gram in the Space of the Analyzed GeneSet

  

Fig 3: KEGG\_GLYCOSPHINGOLIPID\_BIOSYNTHESIS\_GANGLIO\_SERIES: Random ES distribution      
 Gene set null distribution of ES for **KEGG\_GLYCOSPHINGOLIPID\_BIOSYNTHESIS\_GANGLIO\_SERIES**
